# Supplementary material for: Genetic analysis of a potato (Solanum tuberosum L.) breeding collection for southern Colombia using Single Nucleotide Polymorphism (SNP) markers
Source: PLoS One. 2021 Mar 18;16(3):e0248787. doi: 10.1371/journal.pone.0248787 (PMC7971539; doi:10.1371/journal.pone.0248787)
Supplement: Attachment — Submitted filename: Response to Reviewers.pdf [file pone.0248787.s002.pdf]

Dear Editor,

Tzen-Yuh Chiang
Academic Editor
PLOS ONE

Here describe all answers for the editor and reviewers and the changes made in the manuscript,

Commentary editor

1. All references were cited in the list included the suggested by reviewers.
2. The title of the paper was adjusted being the editor commentary: “Genetic analysis of a potato (*Solanum tuberosum* L.) breeding collection for southern Colombia using single nucleotide polymorphism (SNP) markers” in the document and web submission on-line.
3. All manuscript was adjusted being PLOS ONE's style requirements (the format changes as line spacing, tables format and subtitle size was not included in track changes), the author affiliations were corrected because this was inverted to the two last authors.

Commentary reviewers

4. The references suggested was reviewed and included in the manuscript in the lines 340-341 “Others diploid Colombian potato collections have been analyzed using SSR [51] and SNP markers [19] identifying high heterozygosity levels.” and lines 343-346 “The differences between diploid and tetraploid potatoes in the heterozygosity levels has been associated with the ploidy bias, being higher these parameters in polyploid genotypes [53]. However, in this analysis to eliminate this bias all genotypes were analyzed as tetraploids, identifying a minor proportion of heterozygosity levels in diploid genotypes.”. I
5. This, these two new references were included: 51) Juyó D, Sarmiento F, Álvarez M, Brochero H, Gebhardt C, Mosquera T. Genetic Diversity and Population Structure in Diploid Potatoes of Solanum tuberosum Group Phureja. Crop Sci. 2015;55: 760–769. doi:https://doi.org/10.2135/cropsci2014.07.0524 and 53) Bamberg J, del Rio A. Assessing under-Estimation of Genetic Diversity within Wild Potato (Solanum) Species Populations. Am J Potato Res. 2020;97: 547–553. doi:10.1007/s12230-020-09802-3

Additional changes

6. In the text was included: "The genetic materials in this collection belong from multiple germplasm bank origins: the International Potato Center (CIP) of Peru, the Colombian Central Collection (CCC), and the Universidad de Nariño of Colombia and" in the lines 105-107.
7. The sections of Acknowledgments, Funding were edited, while the Contributions section was deleted.
8. Acknowledgments: The authors thank the germplasm banks at the Centro Internacional de la Papa, the Colección Central Colombiana de Papa (AGROSAVIA) of Sistema de Bancos de Germoplasma de la Nación para la Alimentación y la Agricultura (SBGNAA), and the Universidad de Nariño for providing the genetic resources analyzed in this study, the Gobernación de Nariño, Universidad de Nariño and AGROSAVIA for their contribution to the structuring and approval of the project that financed this research.
9. Funding: The resources for the development of this research were provided by the Ministerio de Ciencia y Tecnología de Colombia (Minciencias) and the Gobernación de Nariño through the Fondo de Ciencia, Tecnología e Innovación del Sistema General de Regalias, with the approval of the project "Improvement technological and productive of the potato system in the Department of Nariño", identified with code BPIN No. 2014000100022. The execution of this project was carried out between the Universidad de Nariño and AGROSAVIA through the macro agreement 480-15. The hours spent by researcher Jhon A. Berdugo-Cely MSc. for the development of this study were provided by AGROSAVIA through Variable Transfer (TV) 2019.

Best regards,

Jhon Berdugo,
